# Psychosis Risk is Associated with Increased Multimodal Reports of Socially Salient Content in Degraded Stimuli

**DOI:** 10.1093/schizbullopen/sgag021

**Published:** 2026-05-23

**Authors:** Victor Pokorny, Trevor Williams, Danielle Pratt, Joshua Kenney, Lauren M Ellman, Gregory P Strauss, Elaine F Walker, Scott W Woods, Albert R Powers, Philip R Corlett, Judy L Thompson, Steven M Silverstein, James A Waltz, James M Gold, Jason Schiffman, Vijay A Mittal

**Affiliations:** Department of Psychology, Northwestern University, Evanston, IL 60208, United States; Department of Psychology, Kent State University, Kent, OH 44240, United States; Department of Psychology, Northwestern University, Evanston, IL 60208, United States; Department of Psychiatry, Yale University, New Haven, CT 06519, United States; Department of Psychology & Neuroscience, Temple University, Philadelphia, PA 19122, United States; Departments of Psychology and Neuroscience, University of Georgia, Athens, GA 30602, United States; Department of Psychology and Program in Neuroscience, Emory University, Atlanta, GA 30322, United States; Department of Psychiatry, Yale University, New Haven, CT 06519, United States; Department of Psychiatry, Yale University, New Haven, CT 06519, United States; Department of Psychiatry, Yale University, New Haven, CT 06519, United States; Departments of Psychiatry and Neuroscience, University of Rochester Medical Center, Rochester, NY 14642, United States; Departments of Psychiatry, Neuroscience and Ophthalmology, University of Rochester Medical Center, Rochester, NY 14642, United States; Maryland Psychiatric Research Center, Department of Psychiatry, University of Maryland School of Medicine, Baltimore, MD 21228, United States; Maryland Psychiatric Research Center, Department of Psychiatry, University of Maryland School of Medicine, Baltimore, MD 21228, United States; Department of Psychological Science, 4201 Social and Behavioral Sciences Gateway, University of California, Irvine, CA 92697, United States; Department of Psychology, Northwestern University, Evanston, IL 60208, United States; Institutes for Policy Research (IPR) and Innovations in Developmental Sciences (DevSci), Departments of Psychiatry, Medical Social Sciences, Northwestern University, Evanston, IL 60208, United States

**Keywords:** clinical high-risk, Bayesian inference, hallucinations, predictive coding, perception, perceptual priors

## Abstract

**Background and Hypothesis:**

Humans are capable of detecting faces and speech in noisy environments. While such detection is generally adaptive, we hypothesize that hallucinations and perceptual distortions arise from overdetection of such features under uncertainty.

**Study Design:**

To test this hypothesis, we measured face and speech detection rates in over 800 adolescents and young adults, oversampled for individuals at risk of developing psychotic disorders.

**Study Results:**

Individuals who reported seeing more faces in degraded 2-tone images tended to report hearing more speech in degraded filtered audio clips (*r*(765) = 0.32, *P* < .001). Recognition rates from both tasks independently predicted clinician-rated positive symptoms (face detection: *r*(784) = 0.19, *P* < .001, 95% CI, 0.12-0.26; speech detection: *r*(818) = 0.16, *P* < .001, 95% CI, 0.1-0.23). However, a composite score exhibited a stronger association than either task individually (*r*(760) = 0.23, *P* < .001, 95% CI, 0.16-0.29). This composite score was uniquely predictive of perceptual abnormalities over-and-above other types of positive symptoms such as unusual thought content, suspiciousness, grandiose ideas, and disorganized communication (*b* = 0.066, *t*(781) = 3.01, *P* = .003, partial *r*^2^ = 0.011).

**Conclusions:**

Our results suggest that a supramodal tendency to report the presence of socially salient content under uncertainty may contribute to positive perceptual symptoms and index clinical risk for psychosis. We place these findings within the broader literature and discuss the potential utility of these highly scalable behavioral measures for better identifying individuals at risk for developing psychosis.

## Introduction

Illusory perception of faces and speech has long been observed in both normative and clinical populations.^1–4^ For example, face pareidolia, the perception of illusory faces in everyday objects, is commonly reported in the general population.^4^ In addition to such normative illusory perceptual experiences, voices and faces are common features of overt hallucinations.^1^^,^^3^ In the present article, we argue that atypical perceptual experiences and hallucinations may arise from increased detection of socially salient features under uncertainty. Furthermore, we argue that such increased detection may be an important marker of clinical risk for psychosis.

Prior work has demonstrated altered processing of socially salient stimuli, particularly faces and speech, across phases of psychotic psychopathology.^5–10^ Specifically, the Mooney Faces task is a popular paradigm for studying face processing in chronic schizophrenia,^9^^,^^11^^,^^12^ first-episode,^13^ and clinical high-risk (CHR)^14–16^ populations. Interestingly, while chronic schizophrenia is associated with difficulty integrating local features (ie, perceptual disorganization) leading to a reduced number of face reports,^9–11^ individuals at CHR tend to report seeing more faces.^15^^,^^16^ Thus, performance on the Mooney Faces task varies across clinical stages, possibly due to distinct mechanisms: overweighting of perceptual priors reflecting a vulnerability for the disorder and perceptual disorganization reflecting later dysfunction that accompanies illness onset.

With respect to speech processing, a seminal study from Bentall and Slade^17^ found that hallucination-prone individuals with schizophrenia were more likely to report hearing a voice in white noise than non-hallucination-prone individuals with schizophrenia. More recently, Alderson-Day et al.^18^ found that non-clinical voice hearers recognized speech in degraded audio clips earlier than controls, and before being told that the degraded stimuli contained speech. Using a similar paradigm, Kafadar et al.^6^ reported preliminary findings (19 CHR and 17 control subjects) from the present sample and found that individuals at CHR exhibited higher speech recognition rates than healthy controls. Taken together, the parallel findings of heightened face and speech perception under degraded conditions in CHR samples suggest a supramodal tendency to overweight perceptual priors for socially salient stimuli. However, to our knowledge, no study has directly linked face and speech reports in CHR individuals, nor tested whether a cross-modal index better explains individual variation in positive symptoms generally and perceptual symptoms specifically.

We administered degraded face and speech detection tasks (within the context of a larger battery of tests) to a cohort of adolescents and young adults across a spectrum of clinical risk for psychosis. First, we hypothesized that individuals who reported seeing more faces would also report hearing more speech, consistent with a supramodal tendency toward detection of socially salient objects under uncertainty. Second, we hypothesized that individuals who reported hearing more speech and faces would have more severe positive symptoms, consistent with positive symptoms arising from increased detection of socially salient objects under uncertainty.^6^^,^^15^^,^^17–19^ Third, we hypothesized that reporting more faces and speech would be uniquely related to perceptually-driven positive symptoms such as hallucinations and perceptual distortions as compared to other positive symptoms such as delusions, unusual thought content and disorganized communication. Finally, we hypothesized that individuals that met criteria for a CHR syndrome would be more prone to reporting faces and speech relative to healthy and psychiatric controls. In particular, the comparison between CHR individuals and psychiatric controls can help clarify whether alterations are specific to psychotic psychopathology or are reflective of psychopathology more generally.

## Methods

Data were collected as part of a multisite study of adolescents and young adults at heightened clinical risk for developing psychosis. Recruitment and further study details have been reported elsewhere.^20^ The total sample consisted of 435 CHR, 221 individuals who reported subthreshold positive symptoms, 175 psychiatric controls who did not report any positive symptoms and 246 healthy controls (see Table 1 for demographic information). Participants were recruited through both help-seeking and non-help seeking sources. Participants were considered psychiatric controls if they did not report any positive symptoms, but endorsed a non-psychotic lifetime mental health disorder during the Structured Clinical Interview for DSM-5, Research Version.^21^ Individuals in the CHR group and the subthreshold positive symptom group were allowed to have co-morbid mental health disorders.^22^

**Table 1:** Demographic Information

	**CHR (*n* = 435)**	**SUB (*n* = 221)**	**PCON (*n* = 175)**	**CON (*n* = 246)**	**Statistics**
**Age**	23.3 (4.11)	23.5 (4.16)	23.77 (3.84)	23.53 (4.1)	*F*(3,1051) = 0.57, *P* = .637
**Sex**					X^2^(3) = 2.65, *P* = .448
% Female	63	61	65	58	
% Male	37	39	35	42	
**Race**					X^2^(18) = 62.22, *P* < .001
% African American	15.40	18	12.50	13.50	
% American Indian	0.70	1.40	1.20	0.40	
% Asian	16.30	22.80	24	40.60	
% Caucasian	54.50	50.70	53.60	37.70	
% Hawaiian	0.70	0	0	0.40	
% Multiracial	9.80	5	6.00	6.60	
% Not Reported	2.60	2.30	3.00	0.80	
% Hispanic	16	16	16	10	X^2^(3) = 5.84, *P* = .119
**Education**					X^2^(12) = 14.13, *P* = .293
% High school	62.10	70.80	70.10	68.80	
% Community/2-year college	8.20	6.20	4.10	3.90	
% Four-year college	18.10	17.70	17.50	22.10	
% Graduate school	9.10	3.10	5.20	3.20	
% Other	2.60	2.30	3.10	1.90	
Median household income	$61 000	$70 000	$84 500	$100 000	X^2^(3) = 24.56, *P* < .001

Abbreviations: CHR = clinical high risk group; CON = healthy control group; PCON = psychiatric control group; SUB = subthreshold positive symptom group. Post hoc tests showed that the significant difference in race distribution between groups was primarily driven by a smaller number of Asian participants in the CHR group, a larger number of Asian participants in the CON group and a smaller number of Caucasian participants in the CON group. Significance of group differences in median household income were computed using the Brown-Mood median test. Post hoc median tests of household income revealed the following significant group contrasts: CON-SUB, CON-CHR, SUB-CLN, and PCON-CHR.

CHR status and positive symptom severity was determined according to the Structured Interview for Psychosis-risk Syndromes (SIPS).^23^ Ratings were made by trained raters which included postbaccalaureate and master’s-level research assistants, graduate students, and postdoctoral researchers. Ratings were discussed and evaluated for inter-rater consistency in weekly meetings for each site. Subthreshold status was designated to individuals that did not meet criteria for a CHR status but scored greater than one on any of the SIPS positive subscales.

### Task Details

**Figure 1:**
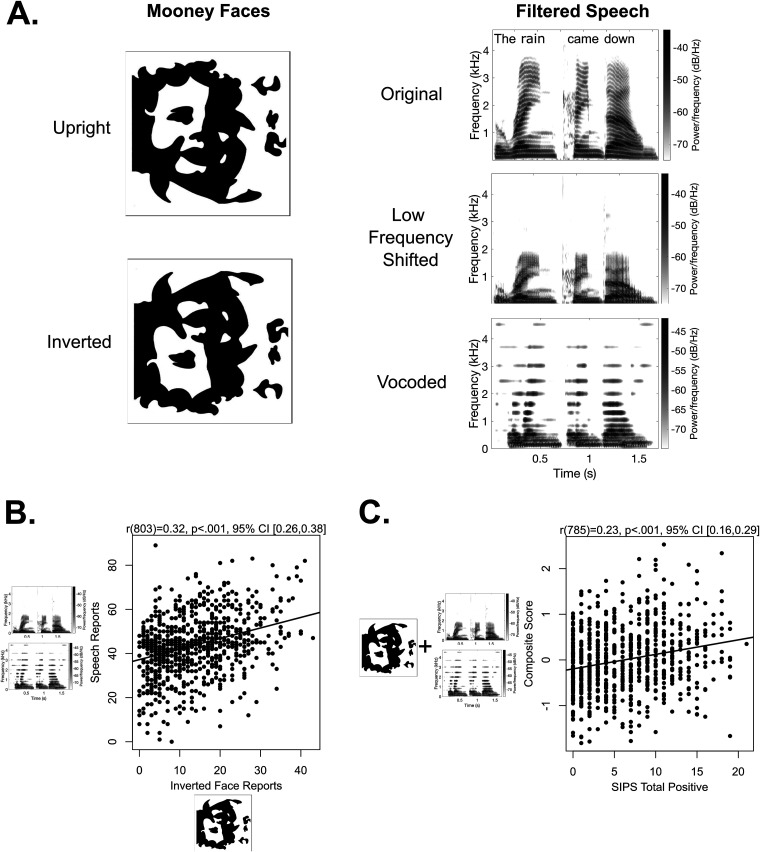
Stimuli Examples and Task Performance Associations. Panel (A) depicts example stimuli from the two tasks. For the face detection task, Mooney face stimuli were presented upright and inverted. For the speech detection task, audio clips of Bamford–Kowal–Bench sentences were pitched-down or vocoded. Panel (B) depicts the association between inverted face reports and speech reports (summed across both pitched-down and vocoded conditions). Panel (C) depicts the association between a composite task score, derived from speech and face recognition rates, and clinician-rated positive symptoms as measured by the Structured Interview for Psychosis-Risk Syndromes (SIPS)

In the Mooney Faces task, 43 black and white images of faces were presented both upright and upside-down (see Figure 1A), pseudorandomly, in a single block. The black-and-white images contained faces of men, women, and children.^24^^,^^25^ For each image, participants were asked to indicate via button press whether they saw a face or not. To assess for response bias, participants were asked to identify, again via button press, the gender (man or woman) and age (adult or child) of each face that they reported seeing. If the subject did not respond within this 5 s time window, the next trial began automatically. Subjects were excluded if they reported seeing more than 10 faces and failed to correctly identify the age or gender of any of the faces they reported seeing which likely indicates a misunderstanding of task instructions. Five subjects were excluded from analysis in this way. We also excluded one individual that did not report seeing a face throughout the entirety of the task.

In the Filtered Speech Recognition task, participants listened to audio clips of a man with a Southern British accent reading Bamford–Kowal–Bench sentences.^26^ There were 3 task conditions (see Figure 1A): a pitched-down condition, a vocoded condition and an unfiltered condition. In the pitched down condition, the stimuli were pitch shifted down one octave. In the vocoded condition, stimuli were filtered using a 16 band vocoder with the carrier frequency bands extending from 100 to 5000 Hz and spaced to produce roughly equal basilar membrane distance.^27^ Participants listened to a total of 45 unique sentences. All 45 sentence clips were pitch-shifted and vocoded such that a total of 135 audio clips (45 unfiltered, 45 pitch-shifted, and 45 vocoded) were presented across the entire task. In the first block, participants were presented with 45 audio clips that were either pitch-shifted or vocoded. Clips were presented in a randomized order and were selected from a pool of all pitch-shifted and vocoded audio clips. This meant that the proportion of pitch-shifted vs. vocoded clips heard in the first block differed across individuals. In the second block, all of the 45 unfiltered sentences were presented in a randomized order. In the third block, all vocoded and pitch-shifted sentences that were not presented in the first block were presented in randomized order. We quantified speech recognition across the first and third blocks, averaging across the 2 conditions. This was done to make the recognition rates from the speech task more comparable with the Mooney Faces recognition rates. However, our primary results held when only analyzing speech rates from the first block (see Supplementary material).

### Analytic Strategy

The primary dependent variables for both tasks were yes/no recognition responses. For the face detection task, we only analyzed the inverted condition because the upright condition tended to be too easy with 50% of subjects reporting seeing more than 80% of the upright faces. For the speech recognition task, we averaged recognition rates across the pitched-down and vocoded conditions.

Bivariate associations between symptom variables and task performance was quantified via Pearson product moment correlations. Results did not change substantially when using Spearman ranked order correlations. We compared the strength of correlations using the cocor.dep.groups.overlap() function from the cocor package^28^ using Pearson and Filon’s z method.^29^ Additionally, given group differences in race and household income (see Table 1), we ran sensitivity analyses including these variables as covariates. None of the primary results were altered by including these covariates (see Supplementary material for more information). To construct a cross-task composite score, we entered the 2 primary task indices (speech reports and inverted face reports) into a unifactor factor analysis model and estimated Thurstone factor scores which maximize the correlation between estimated and true factor scores. For a thorough description of factor scoring methods, see Grice.^30^ With respect to multiple comparisons corrections, we tested a relatively small number of hypotheses per family that were determined a priori such that we report uncorrected *P*-values. However, significance of primary results did not change when adjusting for multiple comparisons using the false discovery rate method (see Supplementary material).

### Transparency Statement

Because the present data are drawn from a large consortium study, some aspects of our results have already been published elsewhere. Group differences in Mooney Faces performance have been reported in subsets of the present sample.^15^^,^^16^ Correlations with functional outcomes have also been reported for the Mooney Faces task.^14^^,^^16^ Pilot data for the filtered speech task (*n* = 17 per group) were reported by Kafadar et al.^6^ The aim of the present paper was to demonstrate that combining scores across tasks in a theoretically grounded way could provide novel insights regarding mechanisms of symptoms. We believe this approach meaningfully extends previous work—which has only looked at these tasks in isolation—and provides novel mechanistic insights.

## Results

### Associations Between Tasks and Symptoms

As depicted in Figure 1B, individuals who reported hearing more speech tended to report seeing more faces (*r*(789) = 0.33, *P* < .001, 95% CI, 0.26-0.39). Additionally, greater clinician-rated positive symptoms, as measured by the Structured Interview for Psychosis-Risk Syndromes,^23^ were associated with more face reports (*r*(809) = 0.19, *P* < .001, 95% CI, 0.12-0.26) and speech reports (*r*(844) = 0.17, *P* < .001, 95% CI, 0.1-0.23). As shown in Figure 1C, when combining face and speech task indices into a composite score, the association with positive symptoms was larger than the association for either task in isolation (*r*(785) = 0.23, *P* < .001, 95% CI, 0.16-0.29). Correlation comparison analyses confirmed that the association between composite score and positive symptoms was significantly larger than the associations for speech reports (*z* = 3.29, *P* = .001) and face reports (*z* = 2.00, *P* = .046) alone.

To assess the role of response bias, we asked participants to identify the gender (man or woman) and age (adult or child) of each face that they reported seeing. When controlling for percentage of correct age/gender judgments as a covariate (lower percentages indicating greater non-perceptual response bias), the association between face reports and positive symptoms remained significant (*b* = 0.101, *t*(795) = 5.12, *P* < .001, partial *r*^2^ = 0.032), as did the association between speech reports and positive symptoms (*b* = 0.06, *t*(773) = 4.47, *P* < .001, partial *r*^2^ = 0.025). Additionally, the number of correct age/gender responses was positively associated with the number of speech reports (*r*(789) = 0.32, *P* < .001, 95% CI, 0.25-0.38). While we cannot definitively rule out response bias effects (see Discussion), this pattern of associations provides convergent evidence that face and speech reports index perceptual processes.

**Figure 2:**
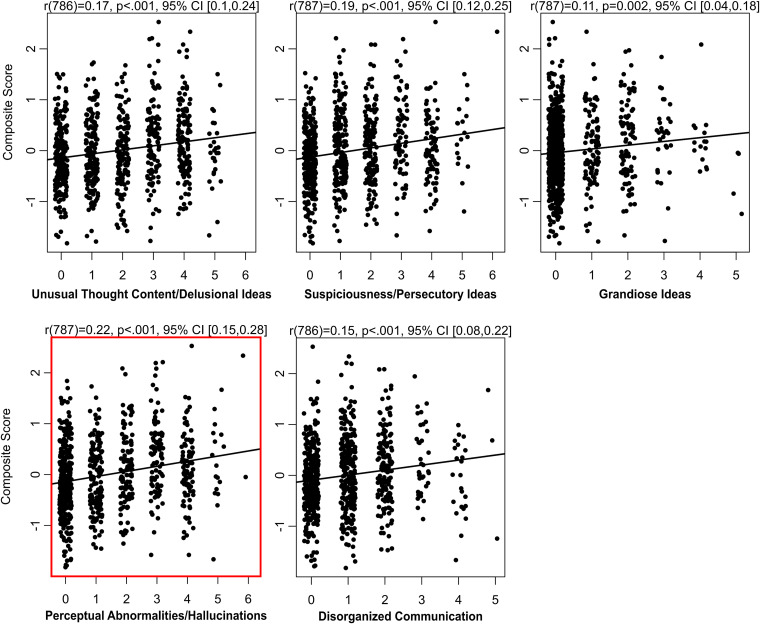
Associations between Composite Index and Positive Symptom Subscales. The composite task index correlated positively with all positive symptom subscales; however, the strongest association was with the perceptual abnormalities/hallucinations subscale (emphasized by thicker border around scatterplot). Horizontal jitter was added to all scatterplots to aid data visualization

While all 5 of the SIPS positive symptom subscales (Unusual Thought Content/Delusional Ideas, Suspiciousness/Persecutory Ideas, Grandiose Ideas, Perceptual Abnormalities/Hallucinations, Disorganized Communication) were positively associated with the composite task score (see Figure 2), the Perceptual Abnormalities/Hallucinations subscale exhibited the strongest association with the composite task score (*r*(787) = 0.22, *P* < .001, 95% CI, 0.15-0.28). While this correlation was numerically larger than correlations with all other subscales, it was only *significantly* larger than the Grandiosity (*z* = −3.08, *P* = .002) and Disorganized Communication (*z* = −2.12, *P* = .034) subscales.

When entering all subscales as predictors in a multiple regression model, the Perceptual Abnormalities/Hallucinations subscale significantly predicted the composite index over-and-above all of the other subscales (*b* = 0.066, *t*(781) = 3.01, *P* = .003, partial *r*^2^ = 0.011). None of the other subscales were significant predictors in this model (see Table 2). This unique association with perceptual positive symptoms provides further convergent evidence that the composite score indexes perceptual processes rather than non-perceptual response biases.

**Table 2:** Multiple Regression of Composite Score on SIPS Positive Symptoms

	*b*	SE	*t*	*P*	Partial *R* ^2^
(Intercept)	−0.191	0.04	−4.82	<.001	0.029
Perceptual abnormalities/hallucinations	0.066	0.022	3.01	.003	0.011
Unusual thought content/delusional ideas	0.006	0.023	0.282	.778	<0.001
Suspiciousness/persecutory ideas	0.04	0.021	1.874	.061	0.004
Grandiose ideas	0.01	0.027	0.37	.711	<0.001
Disorganized communication	0.025	0.028	0.87	.385	0.001

Full model *R*^2^ = 0.1; Adjusted *R*^2^ = 0.055.

### Group and Sex Differences in Task Performance

We also assessed whether the composite task score differed between psychosis risk groups. Our sample consisted of 4 groups: a CHR for psychosis group, a group of individuals who reported subthreshold positive symptoms, a psychiatric control group, and a healthy control group (see Table 1 for demographic information for each group). A one-way ANOVA with the composite score as the dependent variable revealed a significant effect of group (*F*(3,787) = 12.16, *P* < .001). This effect was driven by CHR exhibiting higher composite scores than healthy controls (*t*(501) = 4.99, *P* < .001, Cohen’s *d* = 0.22), psychiatric controls (*t*(436) = 4.2, *P* < .001, Cohen’s *d* = 0.2) and, to a lesser extent, the subthreshold positive symptom group (*t*(476) = 3.38, *P* = .001, Cohen’s *d* = 0.15). Strikingly, when group status and SIPS positive symptom scores were simultaneously entered as predictors into a multiple regression model, positive symptom scores predicted task scores over-and-above group status (*b* = 0.023, *t*(782) = 2.61, *P* = .009, partial *r*^2^ = 0.009) while the group status indicators became insignificant. Thus, the continuously measured SIPS positive score variable was a stronger predictor of task performance than the categorical group indicators. See Figure S1 for a depiction of group differences across all tasks and conditions.

Previous work in this sample has shown that sex assigned at birth may influence group differences in Mooney Faces performance.^15^^,^^16^ However, it is unknown whether a similar effect is present in the supramodal face and speech composite score. To test this, we first assessed the interaction between sex assigned at birth and group status. We did not observe a significant interaction (*F*(3,779) = 0.774, *P* = .509). However, we did observe a general main effect of sex assigned at birth in which males exhibited higher scores than females (*F*(1,779) = 20.682, *P* < .001). Previous meta-analyses^31^ have suggested that samples with higher proportions of male CHR have higher rates of conversion. Thus, it is possible that the observed sex difference in the composite index reflects increased risk in male participants; however, longitudinal follow-up will be necessary to determine whether elevated composite scores prospectively predict conversion.

## Discussion

In the present study, we found that reporting the presence of faces and speech in degraded stimuli was associated with positive symptoms and CHR status in a cohort of over 800 adolescents and young adults. In what follows, we discuss possible mechanisms and implications of these results.

### Bayesian Inference Account of Positive Symptoms

While we cannot rule out alternative interpretations (see next section), we argue that our pattern of results is most consistent with multimodal overweighting of perceptual priors for socially salient content in individuals at CHR. Perception is often formalized as Bayesian inference wherein perceptual hypotheses (ie, priors) and sensory evidence are combined to infer world states. In the context of Bayesian inference, hearing more speech and seeing more faces in degraded stimuli may reflect hyper-precision of a theoretical prior distribution of perceptual hypotheses.^32^ Socially, there is good reason for humans to have strong priors for faces and speech. Perceiving the faces and voices of friends and foes in complex, naturalistic environments is adaptive. However, we argue that there may be a crucial tipping point past which the strength of these priors leads to false inferences in the form of perceptual distortions and hallucinations.^19^

The present study improves upon previous investigations of Bayesian inference in psychotic psychopathology in 3 ways. First, people with psychotic disorders and, to a lesser extent, those at CHR, tend to exhibit generalized cognitive deficits.^33^^,^^34^ Thus, it can be hard to determine whether a case–control difference on a perceptual task is due to something specific about a given task or is due to a generalized impairment. One way to avoid this problem is to identify tasks wherein the clinical group outperforms the control group.^35^ In the present report, we found precisely this: individuals with more positive symptoms tended to identify more faces and speech in the degraded stimuli even after adjusting for accuracy of face reports. Furthermore, by comparing performance to psychiatric controls, we were able to demonstrate that these perceptual alterations were *specific* to psychotic psychopathology rather than due to more general psychopathology factors. This is particularly important because CHR samples tend to exhibit substantial diagnostic comorbidity^22^ that could plausibly influence task performance^36^ and explain group differences between CHR and healthy controls. Second, the multimodal nature of our findings are valuable because they suggest a higher level mechanism may drive previously observed atypical face and speech processing under uncertainty in those at risk for psychosis. Furthermore, the unique link with perceptual symptoms suggests that this supramodal mechanism is perceptually rather than cognitively mediated. Third, previous studies of strength of priors in psychotic psychopathology have exhibited substantial heterogeneity in direction of effects: some studies observe evidence for stronger priors while others observe evidence for weaker priors.^32^ A variety of plausible explanations for this heterogeneity have been offered by others.^32^ However, less discussed is the possibility that this heterogeneity may be, in part, due to small sample sizes of previous work. Small sample sizes can produce large effect sizes by chance in opposing directions when the true effect size is small. This risk lessens as sample size increases. Thus, it is possible that the lack of consistency with respect to direction of effects may be, in part, due to small sample sizes of previous work. By administering highly scalable, computerized assessments to a relatively large number of adolescents and young adults, we are able to provide a more definitive statement on the matter.

### Signal Detection Interpretation of Results

Within the signal detection framework,^37^ the present results are consistent with a more liberal decision criterion in CHR individuals. In the previous section, we interpreted this shift as arising from overweighting of perceptual priors, such that degraded stimuli are more likely to be experienced as faces or speech and consequently endorsed as “yes.” An alternative explanation is that CHR participants simply tended to press “yes” more often irrespective of perceptual experience (ie, a response bias). To address this possibility, in the Mooney Faces task, we included age and gender accuracy checks following face endorsement. If participants were responding indiscriminately, we would expect reduced accuracy on these judgments. However, our primary results remained unchanged when accounting for age and gender accuracy, suggesting that “yes” responses reflected genuine perceptual processing rather than a tendency to respond affirmatively in the absence of an accurate face percept. Moreover, the specific association between perceptual symptom severity and endorsement rates further supports the interpretation that the shift reflects perceptual alterations rather than a non-perceptual response tendency.

In a typical signal detection task, there is a target-absent condition to differentiate discrimination (d’) from decision criterion (c). However, we argue that d’ (z[hits]-z[false alarms]) may not provide a valid index of perceptual overweighting. If perceptual priors are weighted more strongly, ambiguous stimuli may be experienced as faces even when no face is present. In that case, “false alarms” can reflect genuine perceptual experiences relevant to CHR. Indeed, hallucinations and perceptual distortions—core features of psychotic psychopathology—are often conceptualized as false alarms arising from altered perceptual inference. Under this conceptual framework, decision criterion may provide a more direct index of perceptual prior weighting than d′. Still a key limitation of yes/no recognition tasks such as the Mooney faces and Filtered Speech tasks, is that we cannot fully rule out a response bias effect.

### Atypical Processing of Socially Relevant Objects in CHR

The present findings are consistent with previous work demonstrating altered processing of socially salient objects under degraded conditions in CHR individuals. With respect to face processing specifically, work from our own group^15^^,^^16^ and others,^19^ have demonstrated alterations in perception of 2-tone images in CHR. However, while most of this work has used the original Mooney^25^ stimuli, Teufel et al.^19^ instead developed a novel set of stimuli that depicted people rather than faces. Furthermore, the Teufel et al.^19^ paradigm included an exposure manipulation in which the participants saw degraded stimuli, then were exposed to the non-degraded stimuli, and were finally shown the same degraded stimuli again. After exposure, CHR individuals demonstrated better face discrimination (d’) as compared to controls, suggesting a possible perceptual advantage conferred by overweighting of perceptual priors. While our findings cannot provide direct evidence of this effect, they are generally consistent with heightened detection of socially salient objects under degraded conditions in CHR individuals. With respect to perception of degraded speech stimuli, we are aware of only one previous study examining such alterations in CHR individuals. Kafadar et al.^6^ presented preliminary data from the same sample reported in the present study (19 CHR and 17 HC) and, consistent with the present results, observed evidence of increased speech reports in CHR. The unique contribution of the present study to this growing CHR literature is that we directly linked individual differences in degraded face and speech reports and showed that a composite score reflecting across-task performance exhibited better criterion validity (ie, improved prediction of positive symptoms) than either task in isolation. Conceptually, this moves beyond considering these alterations as reflecting distinct, modality-specific mechanisms and instead reframes these alterations as indicators of a supramodal tendency to report socially salient objects under uncertainty.

### Limitations

A limitation of the present work is that we indirectly measured response bias via the percent of correct age and gender responses on the face detection task. One issue with this approach, is that lower percentage of correct age and gender responses may not only reflect response bias, but also could reflect genuine perception of faces that are too vague or unusual for correct identification of age and gender. The present work cannot isolate these 2 possible influences on the age and gender responses. Having said this, controlling for percent of correct responses did not meaningfully influence our primary findings suggesting that the combined influence of response bias and/or vague/unusual face percepts were not strong drivers of our results.

### Conclusion and Future Directions

Better identification of individuals at risk for developing psychotic disorders is crucial for advancing prevention and intervention efforts. The tasks in the present report hold great potential for measuring such risk because they provide a behavioral index of a key component of risk: positive perceptual symptoms. Furthermore, as compared to traditional structured interviews, these tasks are highly scalable and easily accessible to a large number of people. Having said this, the effect sizes of the observed associations were modest. To be clinically useful, these tasks need to be further refined and combined with other tasks that index other aspects of clinical risk for psychosis (eg, negative symptoms, disorganization, cognition, etc.). In this way, degraded face and speech detection tasks may become integral screening tools for identifying individuals at risk of developing psychotic disorders.

## Supplementary Material

Scz_Bull_Open_Supplement_(2)_sgag021
