# Supplementary material for: Psychosis Risk is Associated with Increased Multimodal Reports of Socially Salient Content in Degraded Stimuli
Source: Schizophr Bull Open. 2026 May 23;7(1):sgag021. doi: 10.1093/schizbullopen/sgag021 (PMC13390576; doi:10.1093/schizbullopen/sgag021)
Supplement: Scz_Bull_Open_Supplement_(2)_sgag021 [file Scz_Bull_Open_Supplement_(2)_sgag021.docx]

**Supplemental Materials**

*Effects of Randomly Distributed Trial Proportions*

Because the proportion of pitch-shifted vs. vocoded clips in the first and third blocks differed across individuals, we must consider whether this influenced our primary results. As expected by randomization, there were no differences between groups in proportion of pitch-shifted vs. vocoded trials (F(3,846)=0.39, p=0.76). Similarly, the proportion of pitch-shifted vs. vocoded trials was unrelated to positive symptoms (r(844)=0.02, p=0.563, 95% CI [-0.05,0.09]) and unrelated to the total number of speech reports (r(848)=-0.03, p=0.329, 95% CI [-0.1,0.03]). Furthermore, the association between task performance and positive symptoms was unaffected when controlling for this proportion (b=0.061, t(843)=4.93, p<.001, partial r^2 = 0.028). Taken together, this suggests that differences across individuals in the proportion of vocoded vs. speech trials in a given block are unlikely to explain our primary associations.

*Effects of Collapsing Across Blocks*

When quantifying speech report rates, we collapsed across the first and third blocks. However, it is possible that collapsing across the first and third blocks of the speech task masked interesting group/individual differences in the updating of priors after being exposed to the unfiltered sentences in the second block. Thus, we conducted sensitivity analyses in which we modeled block as a moderating factor. We did not observe a significant group by block interaction (F(3,846)=0.25, p=0.861, η²=<.001) nor a significant positive symptom by block interaction (F(1,844)=0.1, p=0.757, η²=<.001)). This lack of a moderating role of block suggests that collapsing across blocks is a reasonable modeling approach. As a further sensitivity analysis, we assessed whether our main results held when only analyzing the first block. All of our primary results held for such analyses. For example, we observed significant associations between first block speech reports and inverted face reports (r(789)=0.28, p<.001, 95% CI [0.21,0.34]) and positive symptoms (r(844)=0.15, p<.001, 95% CI [0.08,0.22]).

*Full Sample vs. CHR Only Associations*

The analyses reported in the main text examined associations across groups. This approach maximized power by increasing sample size and included the broadest range of behavioral and symptom scores. However, it is possible that collapsing across groups masked interesting within-group associations, particularly with respect to the CHR group. Thus, we recomputed the main results for CHR only as a follow-up analysis. The correlation between speech and face recognition rates remained significant within the CHR group only (r(312)=0.33, p<.001, 95% CI [0.22,0.42]). The correlation between positive symptoms and speech reports remained significant (r(328)=0.13, p=0.016, 95% CI [0.02,0.24]); however, the correlation between positive symptoms and face reports did not (r(324)=0.04, p=0.479, 95% CI [-0.07,0.15]). Consequently, the correlation between the composite score and the positive symptoms fell to a trend level (r(312)=0.1, p=0.08, 95% CI [-0.01,0.21]). However, the correlation between the composite score and the Perceptual Abnormalities/Hallucinations subscale remained significant (r(312)=0.13, p=0.018, 95% CI [0.02,0.24]). Furthermore, when entering all subscales as predictors in a multiple regression model, we again found that the Perceptual Abnormalities/ Hallucinations subscale significantly predicted the composite score over-and-above the other subscales (b=0.063, t(308)=2.14, p=0.033, partial r^2 = 0.015).

Thus, we were able to recapitulate a number of the full sample findings within the CHR group only. For the associations that did not remain significant when subsetting to CHR, there are a few possible explanations. First, the sample size is considerably reduced when computing associations within the CHR group such that larger effect sizes are required to obtain significance. Second, the range of symptom scores is reduced within the CHR group compared to the entire sample because one must score above a certain threshold to be considered CHR. Restricting the range of variables is known to attenuate the strength of bivariate associations. Third, it is possible that between group differences are important drivers of the full sample associations.

*Adjusting for Race and Income*

Given the group differences in race and household income, we also ran sensitivity analyses controlling for these variables. For correlational analyses, we adjusted for these covariates using multiple regression, entering race as a categorical variable and household income as a log transformed continuous variable. The association between inverted face reports (entered as dependent variable) and speech reports (entered as predictor) remained highly significant when controlling for these variables (b=0.221, t(759)=9.53, p<.001, partial r^2 = 0.107). Similarly, the association between speech reports and positive symptoms (b=0.437, t(814)=4.48, p<.001, partial r^2 = 0.024) and the association between inverted face reports and positive symptoms (b=0.352, t(775)=5.35, p<.001, partial r^2 = 0.036) remained significant. The association between the composite score and positive symptoms also remained significant (b=0.031, t(755)=6.17, p<.001, partial r^2 = 0.048) and was greater than either task association in isolation. The Perceptual Abnormalities/Hallucinations subscale remained significantly associated with the composite score (b=0.095, t(757)=5.62, p<.001, partial r^2 = 0.04). Importantly, the key finding that the Perceptual Abnormalities/Hallucinations subscale predicted composite scores over-and-above the other subscales was robust to adjusting for race and income (b=0.057, t(751)=2.5, p=0.013, partial r^2 = 0.008). Finally, the effect of group status on the composite score remained significant when adjusting for these variables (F(3,757)=10.246, p<.001) with all reported post hoc comparisons remaining significant (ps<.01).

*Adjustment for Multiple Comparisons*

In the main text, we report uncorrected p-values because we tested a relatively small number of hypotheses that were determined a priori. To demonstrate that conclusions do not depend on this decision, we also report false discovery rate (FDR) correction within families of related tests [(Benjamini and Hochberg 1995)](https://paperpile.com/c/zlpfCU/n8cy). The family of tests with the greatest potential for inflated Type I error was the five associations between the composite score and the SIPS positive symptom subscales. All of these associations remain significant after correction (FDR-adjusted p-values: Unusual Thought Content/Delusional Ideas, p < .001; Suspiciousness/Persecutory Ideas, p < .001; Grandiose Ideas, p = .003; Perceptual Abnormalities/Hallucinations, p < .001; Disorganized Communication, p < .001). Additionally, following the significant omnibus ANOVA effect of the group on composite score, post hoc comparisons between the CHR group and the three non-CHR groups remained significant after false discovery rate correction (all FDR-adjusted p < .001). Sensitivity analyses were not corrected for multiple comparisons, as they were conducted to assess robustness of primary findings rather than to test additional hypotheses.


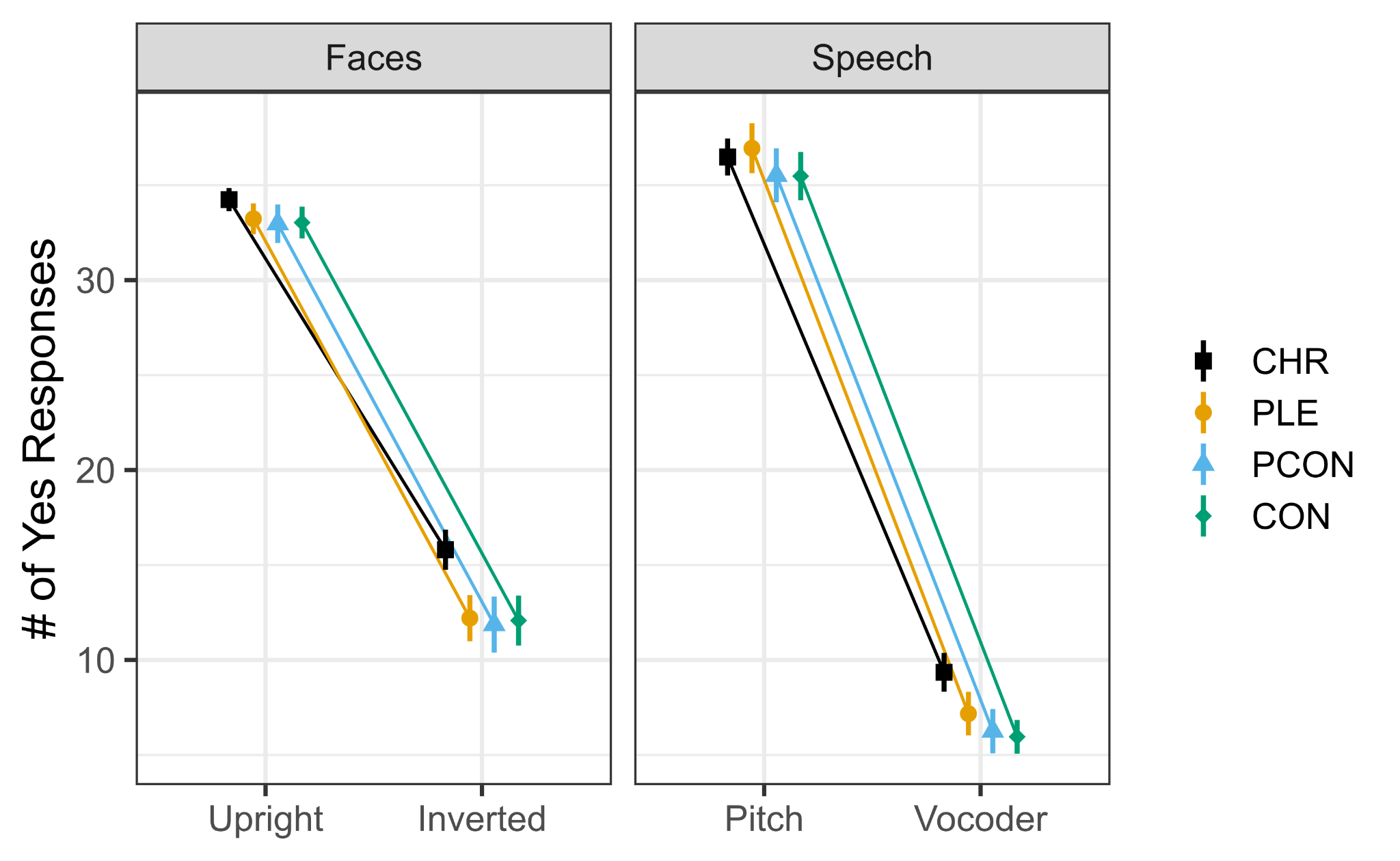


**Supplemental Figure 1. Performance across all conditions, groups and tasks.** Conditions are labeled along the x-axis with the more ambiguous condition on the right for each task. For both tasks, CHR tended to differ most from the other groups for the more difficult condition in both tasks. Consistent with this, we observed a significant group by difficulty interaction across task scores (F(3,786)=11.6, p<.001, η²=.042).
